# Inhibition of soluble epoxide hydrolase enhances the dentin-pulp complex regeneration mediated by crosstalk between vascular endothelial cells and dental pulp stem cells

**DOI:** 10.1186/s12967-024-04863-y

**Published:** 2024-01-16

**Authors:** Lingwenyao Kong, Juanjuan Li, Yuwen Bai, Shaoyang Xu, Lin Zhang, Weixian Chen, Lu Gao, Fu Wang

**Affiliations:** 1 School of Stomatology, Dalian Medical University, No. 9 West Section, Lvshun South Road, Dalian, 116044 People’s Republic of China; 2 Academician Laboratory of Immune and Oral Development & Regeneration, Dalian Medical University, Dalian, China; 3 The Affiliated Stomatological Hospital of Dalian Medical University, Dalian, China

**Keywords:** TPPU, DPSCs, Pulp regeneration, Vascularization, Angiogenic-odontogenic coupling, HIF-1α, TGF-β, VEGFR2

## Abstract

**Background:**

Revascularization and restoration of normal pulp-dentin complex are important for tissue-engineered pulp regeneration. Recently, a unique periodontal tip-like endothelial cells subtype (POTCs) specialized to dentinogenesis was identified. We have confirmed that TPPU, a soluble epoxide hydrolase (sEH) inhibitor targeting epoxyeicosatrienoic acids (EETs) metabolism, promotes bone growth and regeneration by angiogenesis and osteogenesis coupling. We hypothesized that TPPU could also promote revascularization and induce POTCs to contribute to pulp-dentin complex regeneration. Here, we in vitro and in vivo characterized the potential effect of TPPU on the coupling of angiogenesis and odontogenesis and investigated the relevant mechanism, providing new ideas for pulp-dentin regeneration by targeting sEH.

**Methods:**

In vitro effects of TPPU on the proliferation, migration, and angiogenesis of dental pulp stem cells (DPSCs), human umbilical vein endothelial cells (HUVECs) and cocultured DPSCs and HUVECs were detected using cell counting kit 8 (CCK8) assay, wound healing, transwell, tube formation and RT-qPCR. In vivo, Matrigel plug assay was performed to outline the roles of TPPU in revascularization and survival of grafts. Then we characterized the VEGFR2 + POTCs around odontoblast layer in the molar of pups from C57BL/6 female mice gavaged with TPPU. Finally, the root segments with DPSCs mixed with Matrigel were implanted subcutaneously in BALB/c nude mice treated with TPPU and the root grafts were isolated for histological staining.

**Results:**

In vitro, TPPU significantly promoted the migration and tube formation capability of cocultured DPSCs and HUVECs. ALP and ARS staining and RT-qPCR showed that TPPU promoted the osteogenic and odontogenic differentiation of cultured cells, treatment with an anti-TGF-β blocking antibody abrogated this effect. Knockdown of HIF-1α in HUVECs significantly reversed the effect of TPPU on the expression of angiogenesis, osteogenesis and odontogenesis-related genes in cocultured cells. Matrigel plug assay showed that TPPU increased VEGF/VEGFR2-expressed cells in transplanted grafts. TPPU contributed to angiogenic-odontogenic coupling featured by increased VEGFR2 + POTCs and odontoblast maturation during early dentinogenesis in molar of newborn pups from C57BL/6 female mice gavaged with TPPU. TPPU induced more dental pulp-like tissue with more vessels and collagen fibers in transplanted root segment.

**Conclusions:**

TPPU promotes revascularization of dental pulp regeneration by enhancing migration and angiogenesis of HUVECs, and improves odontogenic differentiation of DPSCs by TGF-β. TPPU boosts the angiogenic–odontogenic coupling by enhancing VEGFR2 + POTCs meditated odontoblast maturation partly via upregulating HIF-1α, which contributes to increasing pulp-dentin complex for tissue-engineered pulp regeneration.

**Supplementary Information:**

The online version contains supplementary material available at 10.1186/s12967-024-04863-y.

## Background

Dental pulp is a highly vascularized soft tissue with various physiological functions, including sensory, defense, nutrition, and repair functions, which of the foundation is good microcirculation [1, 2]. The local trauma, caries, and periodontal disease lead to pulp or periapical disease. Traditional care options include root canal therapy or apexification. However, due to the loss of the dental pulp, the tooth loses the function of immune protection, sensitivity, and formation of secondary dentin, which increases the risk of fracture and discoloration [3, 4]. For some immature permanent teeth, the formation of the apical foramen may be stagnant due to infection or trauma. The traditional apical induction method cannot obtain normal apical structure and dental pulp tissue, resulting in unsatisfactory long-term efficacy [5]. How to eliminate inflammation and restore healthy pulp has been a hot spot in the field of dentistry. With the development of regenerative medicine, tissue engineering-based dental pulp regeneration has shown great promise. The stem cell-based tissue engineering technology achieves significant progress by replacing the diseased dental pulp, making dental pulp regeneration possible. The special anatomical structure of pulp cavity, including narrow root canal space and small apical foramen, limited blood supply origin and restricted pulp regeneration. Therefore, it is vital to find chemicals that can promote tissue regeneration and revascularization for pulp regeneration.

Epoxyeicosatrienoic acids (EETs) are fatty acids with various biological activities that are synthesized from arachidonic acid (AA) by cytochrome P450 (CYP) and are rapidly hydrolyzed by soluble epoxide hydrolase (sEH) to corresponding dihydroxyeicosatrienoic acids (DHETs) [6, 7]. Recent studies have shown that EETs have multiple biological effects, such as anti-inflammatory and analgesic, inhibiting adipogenic differentiation of stem cells, enhancing angiogenesis, and promoting tissue growth and organ regeneration [6, 8]. In cardiovascular system, EETs modulate coronary vessels to increase myocardial blood flow [9]. In nervous system, EETs can increase cerebral blood flow, inhibit neuronal apoptosis, reduce neuroinflammation, attenuate brain injury and promote neurological recovery [10]. In addition, EETs can also inhibit adipogenesis of mesenchymal stem cells (MSCs) and preadipocytes [11]. Considering the multiple biological effects of EETs, people began to apply them in the field of tissue engineering to explore their effects on tissue regeneration. Panigrahy et al. found that liver regeneration proceeds faster in transgenic mice with high expression of EETs after partial hepatectomy than in wild-type mice. Exogenous systemic administration of EETs also promotes liver regeneration in mice [12]. Our previous experiments also confirmed that EETs enhance the formation of new bone, collagen fibers and vascular structures in a mouse calvarial defect model [13]. However, it has not been reported whether EETs can promote the coupling of angiogenesis and odontogenesis, thereby promoting pulp tissue regeneration.

The key to regenerating tissue-engineered pulp is to promote the formation of a vascular network in the graft, and early blood flow reconstruction is necessary to provide sufficient oxygen and nutrients to the regenerating tissue [14]. HIF-1α/VEGF is an important regulatory pathway that regulates angiogenesis. The VEGF family consists of six protein isoforms called VEGF-A, VEGF-B, VEGF-C, VEGF-D, VEGF-E, VEGF-F and placental growth factor-1 and -2 (PIGF). VEGF binds to three members of the transmembrane tyrosine kinase receptor VEGFR, VEGFR1 and VEGFR2, which have high affinity for VEGF-A. VEGFR3 has specificity for VEGF-C and VEGF-D [15]. Binding to the ligand VEGF-A, VEGFR-2 mediates endothelial cell proliferation, invasion, migration and survival through the MAPK signaling pathway [16, 17]. Matsubara T et al. identified a vessel in the dental pulp that promotes dentin differentiation, named the POTCs vessel, which differs from the Type H vessel in the skeletal system and is highly expressed in VEGFR2 and VEGFR1, and also demonstrated that VEGFR2 is a key factor in angiogenesis-dentinogenic coupling [18].

In this work, we build an in vivo transplantation system for DPSCs and root canal complex and in vitro coculture system to explore that TPPU, a soluble epoxide hydrolase inhibitor (sEHi), promotes early vascularization of tissue-engineered pulp regeneration and DPSCs odontoblast differentiation, further clarify the mechanism and feasibility of TPPU in tissue-engineered pulp regeneration, and provide a theoretical basis for targeting sEH to promote pulp regeneration.

## Methods

### Chemicals

TPPU (MedChemExpress, NJ, USA) was dissolved in DMSO (10 mg/ml) and stored at − 20 °C according to the manufacturer’s instructions. TPPU stock solution was added into medium (the final concentration of TPPU was 10 μM) for in vitro cell treatment as previously described [13, 19]. For in vivo study, TPPU stock solution (10 mg/ml dissolved in DMSO) was formulated in 20% (vol/vol) PEG 400 (MedChemExpress, the final concentration of TPPU was 1 mg/ml). The mice were administered with TPPU in 20% PEG400 by oral gavage (3 mg/kg) every other day for 2 weeks as previously described [20–22].

### Animals

BALB/c nude mice and C57BL/6 mice were obtained from the Animal Experimental Center of Dalian Medical University and randomly assigned into TPPU-treated and control groups. All animals were kept at room temperature 22 °C, humidity 50%-60%, and a 12 h light–dark cycle. All animal procedures were conducted and reported according to ARRIVE (Animal Research: Reporting of in Vivo Experiments) guidelines and were approved by the Institutional Animal Care and Use Committee of Dalian Medical University (No. AEE22005, No. AEE22004). The sample size was decided according to the guidelines for sample size calculations of Boston University. If the animal died prematurely, preventing the collection of histological data (no died prematurely in our study).

### Cell culture

Human dental pulp stem cells (DPSCs) were isolated from third molar extractions of patients from the Affiliated Stomatological Hospital of Dalian Medical University and cultured as described previously [23]. All operations were in accordance with the requirements of the Ethics Committee of the Affiliated Stomatological Hospital of Dalian Medical University (NO.2022001). Informed consent was obtained from all participants. Human umbilical vein endothelial cells (HUVECs, ATCC, Manassas, VA, USA) were cultured in endothelial cell medium (ECM, ScienCell, California, USA) with 5% fetal bovine serum (FBS, Gibco, Grand Island, USA), 1% penicillin–streptomycin (Gibco), and 1% endothelial cell growth supplement (ECGS, ScienCell) at 37 °C in a humidified atmosphere with 5% CO_2_.

### Cell proliferation

DPSCs, HUVECs, and cocultured DPSCs and HUVECs (1:1 ratio) were seeded in 96-well plates (8 × 10^3^ cells/well). The experimental groups were treated with TPPU (10 μM). After culturing for 1 day, 3 day, and 5 days, the cells were performed for cell proliferation assays using a Cell Counting Kit-8 (CCK-8, Biotech, Tianjin, China). Briefly, 10 μl of CCK8 reagent was added to each well and then incubated at 37 °C for 1 h, and the absorbance at 450 nm was measured with a microplate reader (Bio-Rad, California, USA).

### Wound healing assay

DPSCs, HUVECs, and cocultured DPSCs and HUVECs (1:1 ratio) were plated at 1 × 10^5^ cells/well in 6-well plates and treated with or without TPPU (10 μM) until 80% confluence, respectively. A scratch across the center of the monolayer was made using a 200 μl pipette tip. After gently washing the wells twice, the cells were cultured in a medium with TPPU (10 μM) or vehicle. The area of scratch was photographed at 0 h and 24 h, and the width of the wound area was quantitatively evaluated using ImageJ software (National Institutes of Health, NIH, USA).

### Transwell migration assay

A total of 5 × 10^5^ HUVECs per well were plated in Transwell insert, and DPSCs were inoculated in the lower chamber at 5 × 10^5^ cells/well. After the cells were treated with or without TPPU (10 μM) for 24 h, the upper side of the insert membrane was cleansed with a cotton swab to remove the cells that had not passed through the membrane. The cells on the lower surface of the insert membrane were fixed with 4% paraformaldehyde (PFA) for 15 min and stained with 0.1% crystal violet for 30 min. The number of migrating cells was manually counted under the inverted microscope (IX71, Olympus, Japan) and statistically analyzed.

### Tube formation assay

DPSCs, HUVECs or cocultured HUVECs and DPSCs (1:1 ratio) pretreated with/without TPPU (10 μM) for 3 day were seeded at the density of 2 × 10^4^/well onto Matrigel-coated 96-well plate, respectively and then incubated for 6 h. The tube formation was observed under an inverted microscope. The number of tubes was determined using AngioTool software (NIH, USA).

### Osteogenic differentiation

Cocultured HUVECs and DPSCs (1:1 ratio) were seeded at a density of 2 × 10^4^ cells/well into a 12-well plate. Osteogenic differentiation was induced using osteogenic induction medium containing α-MEM with 10% FBS, 1% penicillin–streptomycin, 50 mg/l ascorbate 2-phosphate, 0.1 μM dexamethasone, and 10 mM β-glycerophosphate disodium. During the osteogenic induction period, cells were treated with/without TPPU (10 μM) for 7 days or 21 days. The medium was changed every 3 day. After 7 days or 21 days of induction, cells were fixed by 4% PFA and stained with Alkaline phosphatase staining Kit (ALP, Sigma-Aldrich, USA) and Alizarin red staining (ARS, Sigma-Aldrich, USA) according to the manufacturer's procedure, respectively.

### Cell transfection

The HIF-1α knockdown virus was purchased from GiKaiGene (Shanghai, China), and when the cell density reached 50% according to MOI (1:20) for virus infection, the pro-infection reagent (HistransPG, 1:24 diluted with complete medium) was added for 16 h. The infection efficiency was observed after 72 h, and cell screening was performed using 0.1% puromycin (Coolaber, Beijing, China). RT-qPCR and Western Blotting were used to detect the knockdown efficiency.

### Real-time quantitative reverse transcription polymerase chain reaction (RT-qPCR)

Total RNA was extracted from the cells with TRIzol^®^ reagent (Invitrogen, Carlsbad, CA, USA) according to the manufacturer’s instructions. Total RNA (1 μg) was reverse transcribed using HiScript II Reverse Transcriptase (Vazyme, Nanjing, China). RT-qPCR was performed using SYBR Green PCR Master Mix (Vazyme) according to the manufacturer’s instructions by TP800 (Takara, Otsu, Japan). Relative mRNA expression was calculated after normalization to the housekeeping *GAPDH* gene using the 2^−ΔΔCt^ method. The primer sequences are listed in Additional file 4: Table S1.

### Dentin discs for cell culture

The extracted third molars in the Affiliated Stomatological Hospital of Dalian Medical University were selected to prepare a 1.5 mm thick dentin disc with a slow-speed dental handpiece under water cooling. All operations were in accordance with the requirements of the Ethics Committee of the Affiliated Stomatological Hospital of Dalian Medical University (No. 2022001). Informed consent was obtained from all participants included in the study. The dentin discs were cleaned and sterilized according to a previous study [24]. Briefly, the dentin discs were sequentially rinsed and ultra-sonicated in 10% EDTA for 30 min, 3% NaClO for 30 min, 3% hydrogen peroxide for 30 min, and PBS 3 times for 15 min each time. The cleaned dentin discs were placed in 75% alcohol for 24 h, then soaked in PBS containing 2% penicillin–streptomycin and stored aseptically at 4 °C. The dentin discs were exposed to UV light overnight before the experiment. DPSCs and HUVECs (1:1) were inoculated on the dentin discs treated with TPPU (10 μM) for 7 d and were collected.

### In vivo Matrigel plug assay

The male BALB/c nude mice (8 weeks of age) were randomly divided into nude mice + PEG400 group (TPPU- group, n = 6); and nude mice + PEG400 + TPPU group (TPPU + group, n = 6). The TPPU + group was orally gavaged with TPPU (3 mg/kg) every other day for 2 weeks (from 1 week before procedure through 1 week after procedure). The TPPU- group was orally gavaged with only PEG400. Matrigel plugs (80 μl) including Matrigel alone; Matrigel with DPSCs (1 × 10^7^/ml) and Matrigel with DPSCs and HUVECs (1:1, 1 × 10^7^/ml) were slowly subcutaneously injected into both sides of the back of the BALB/c nude mice under anesthesia with an intraperitoneal injection of ketamine (100 mg/kg, VetaKetam, Poland) and xylazine (10 mg/kg, Sedasin, Biowet, Poland). After 1 week, Matrigel plugs were isolated for histology.

### Tissue-engineered pulp regeneration

Human mandibular first premolars extracted for orthodontic treatment were collected from the Affiliated Stomatological Hospital of Dalian Medical University with informed consent. The roots of mandibular first premolars were sectioned into 5 mm long segments. Root canals were shaped with a diameter of 2 mm using rotary instruments, then cleaned and sterilized as described previously. The Matrigel mixed with DPSCs (1 × 10^7^/ml, 200 μl/root) were injected into the prepared root canals and incubated for 2 h at 37 °C, then the root fragments were implanted subcutaneously into the dorsum of male BALB/c nude mice under anesthesia (2 root segments/mouse). The male BALB/c nude mice (8 weeks of age) were randomly divided into nude mice + PEG400 group (TPPU- group, n = 5); nude mice + PEG400 + TPPU group (TPPU + group, n = 5). The BALB/c nude mice in TPPU + group were orally gavaged with TPPU in PEG400 (3 mg/kg) every other day for 2 weeks (from 1 week before procedure through 1 week after procedure), and the BALB/c nude mice in TPPU- group were orally gavaged with PEG400 alone every other day for 2 weeks. The root fragments were harvested 8 weeks after transplantation, fixed with 4% PFA for 24 h, demineralized with 10% EDTA for 8 weeks, then further prepared for histological analysis.

### Newborn pups handling

The C57BL/6 mice (6 weeks of age) were mated and the detection of a vaginal plug the following day morning was considered to be gestational day 0.5. The pregnant mice were orally gavaged with or without TPPU in PEG400 (3 mg/kg) from gestational day 13.5 through to postnatal day 7 (P7). The newborn pups were sacrificed at P12. The mouse heads were fixed in 4% PFA. The lower jawbones were dissected, decalcified in 10% EDTA, and embedded in paraffin. Frontal serial Sects. (6 μm) were cut for immunohistochemical staining.

### Histological staining and immunostaining

The isolated tissues were prepared for paraffin embedding and paraffin Sects. (6 μm). Histological hematoxylin and eosin (H & E, Solarbio, Beijing, China) staining and Masson’s trichrome staining (Solarbio) were performed according to the manufacturer's protocol [25].

For immunohistochemistry (IHC) staining, sections were incubated with the primary antibody rabbit polyclonal anti-CD31 (ab28364, 1:50, Abcam), rabbit monoclonal anti-HIF-1α (ab2185, 1:200, Abcam), mouse monoclonal anti-Ki-67 (ab279653, 1:200, Abcam), rabbit polyclonal anti-DSPP (ab122321, 1:100, Abcam), rabbit polyclonal anti-TGF-β (ab31013, 1:50, Abcam) and rabbit polyclonal anti-VEGFR2 (26,415-1-AP, 1:200, Proteintech, Wuhan, China) overnight at 4 °C after antigen retrieval and blocking procedures. Then, sections were incubated with a universal two-step detection kit (PV-9000, ZSGB-BIO, Beijing, China), and horseradish-labeled chain ovalbumin for 15 min (ZSGB-BIO) at room temperature, followed by 3,3-diaminobenzidine (DAB, ZSGB-BIO) as chromogen and hematoxylin as counterstain.

For immunofluorescence (IF), the primary antibodies were used by rabbit polyclonal anti-VEGFR2 (26,415-1-AP, 1:200, Proteintech), rabbit monoclonal anti-VEGF (ab52917, 1:200, Abcam) and mouse monoclonal anti-EMCN (ab106100, 1:200, Abcam), the secondary antibodies were used by goat anti-rat IgG (Dylight 549, A23340-1, Abbkine, California, USA), goat anti-rabbit IgG (Dylight 488, A23220, Abbkine), and nuclear staining was performed using antifade mounting medium with DAPI (Coolaber). Quantitative analysis by image-pro plus software (Media Cybernetics, USA).

### Western blotting

Protein extraction was followed by concentration determination, referring to the previous description [26]. The PVDF membrane containing protein was blocked with 5% milk, incubated with the primary antibody of rabbit monoclonal anti-HIF-1α (ab2185, 1:1000, Abcam), rabbit polyclonal anti-TGF-β (ab31013, 1:1000, Abcam) and rabbit polyclonal anti-VEGFR2 (26,415-1-AP, 1:1000, Proteintech) at 4 °C overnight and the secondary horseradish peroxidase antibody (ZB2301, ZSGB-BIO) for 1 h at room temperature, and then identified using the high-sig ECL kit (Tanon, Shanghai, China). Bio-Rad VersaDoc image system (Bio-Rad) was used for detection and statistical analysis.

### Statistical analysis

Data were presented as the mean ± SEM and analyzed with SPSS 25.0 software (IBM, USA). An unpaired Student’s t-test was used for comparison of two groups. One-way analysis of variance (ANOVA) and Tukey’s post hoc test was performed for comparison of three or more groups. Statistical significance was defined as *P* < 0.05.

## Results

### TPPU enhances osteogenic and odontogenic differentiation of cocultured cells via upregulating TGF-β

**Fig. 1:**
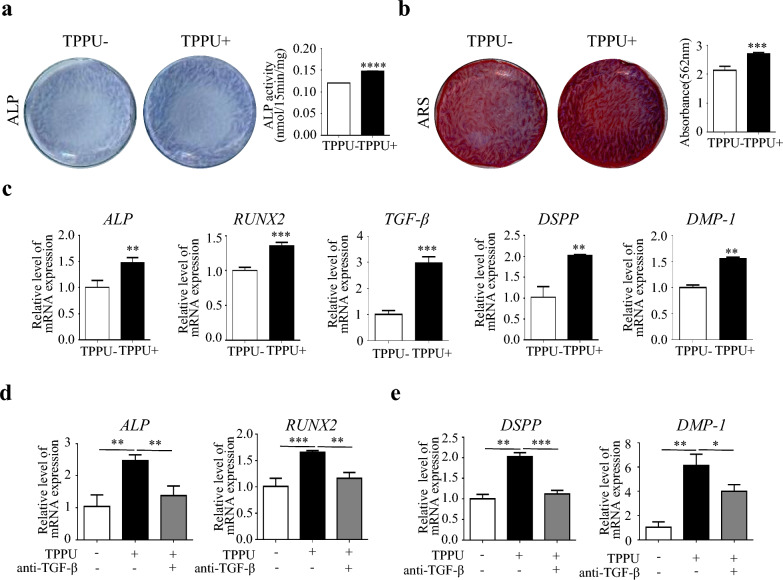
TPPU promotes osteogenic/odontogenic of cocultured HUVECs and DPSCs in vitro via TGF-β **a** Representative images of ALP staining after 7 days of osteogenic induction. **b** Representative images of ARS staining after 21 days of osteogenic induction. **c** RT-qPCR showed the expression of the osteogenic/odontogenic related gene of *ALP, RUNX2*, *TGF-β, DSPP* and *DMP-1* after 7 days of osteogenic induction. **d**, **e** The expression of TGF-β in DPSC was blocked with a neutralization antibody, and the mRNA expression levels of *ALP, RUNX2*, *DSPP*, and *DMP-1* were detected by RT-qPCR after 7 days of osteogenic induction. *P < 0.05, **P < 0.01, ***P < 0.001, ****P < 0.0001

The ECs promote the osteogenic differentiation of DPSCs in direct co-cultures. However, whether TPPU can promote odontogenic differentiation via ECs has not been demonstrated. In this study, ALP and ARS staining showed that TPPU could significantly promote the osteogenic differentiation of cocultured cells (Fig. 1a, b, P < 0.001). RT-qPCR results showed that TPPU upregulated the expression of *ALP* and *RUNX2* (Fig. 1c, P < 0.01). Next, we investigated whether TPPU could further improve the odontogenic differentiation potential, showing the expression of *TGF-β, DPSS*, and *DMP-1* associated with odontogenic differentiation was increased significantly in TPPU-treated cocultured cells (Fig. 1c, P < 0.01). While TPPU has no effects on osteogenic differentiation of DPSCs alone in our previous study, implying that TPPU could promote osteogenic and odontogenic differentiation through enhanced crosstalk between DPSCs and HUVECs. Since the odontogenic differentiation of DPSCs is vital for dental pulp regeneration and TGF-β plays a critical role in epithelial-mesenchymal interactions and odontoblast maturation, Western blotting also confirmed that TPPU treatment significantly increased TGF-β expression in the cocultured cells (Additional file 2: Fig. S2a). We next detected the role of TGF-β in TPPU-enhanced odontoblastic differentiation of cocultured cells. When TGF-β was blocked using a neutralization antibody, the ability of TPPU to improve odontogenic and osteogenic differentiation of cocultured cells was attenuated (Fig. 1d, e, P < 0.05). These results demonstrated that TPPU-treated cocultured cells were more susceptible to odontogenic and osteogenic differentiation partly via upregulating TGF-β.

### TPPU promotes migration and angiogenesis of the cocultured HUVECs and DPSCs in vitro

**Fig. 2:**
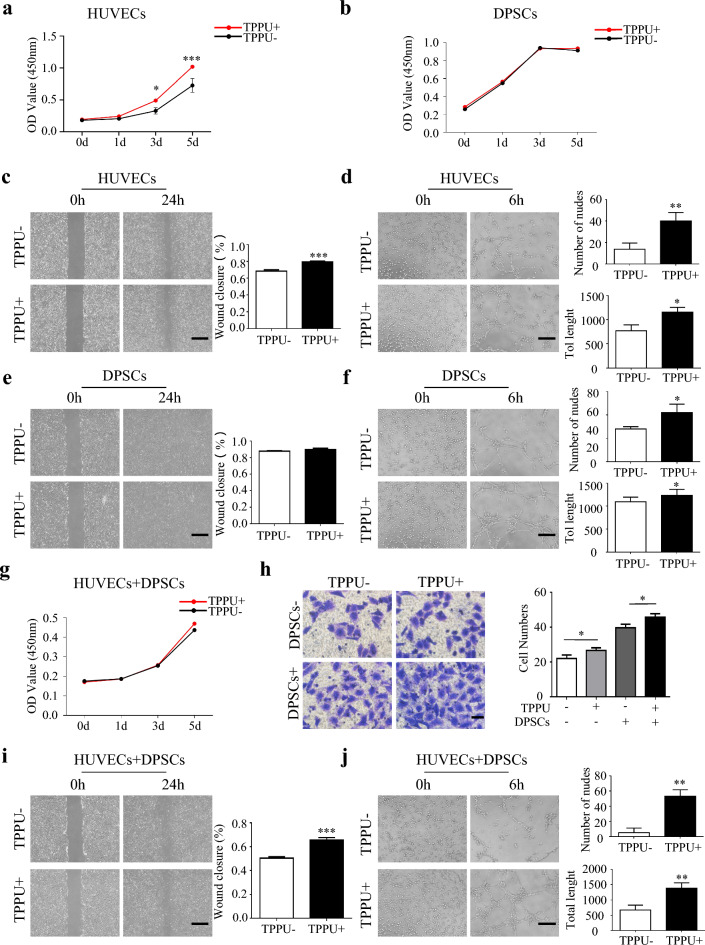
The effect of TPPU on proliferation, migration, and angiogenesis of HUVECs and DPSCs **a**, **b** Quantitative analysis of CCK8 on days 0, 1, 3, and 5 after TPPU treatment in HUVECs or DPSCs. **c**, **e** Representative images of the scratch assay in HUVECs and DPSCs treated with TPPU after 24 h, scale bar = 500 µm. And quantitative analysis of the percentage of wound closure. **d**, **f** Representative images of tube formation in HUVECs and DPSCs treated with TPPU after 6 h, scale bar = 100 µm. And quantitative analysis of total length and the number of nudes in TPPU + and TPPU- group. **g** Quantitative analysis of CCK8 on days 0, 1, 3, and 5 after TPPU treatment in coculture of HUVECs and DPSCs. **h** Representative image of transwell assay at 24 h after TPPU treatment, scale bar = 50 µm. And quantitative analysis of the migration cell numbers. **i** Representative images of scratch assay in coculture of HUVECs and DPSCs at 24 h after TPPU treatment, scale bar = 500 µm. And quantitative analysis of the percentage of wound closure. **j** Representative images of tube formation in coculture of HUVECs and DPSCs at 6 h after TPPU treatment, scale bar = 100 µm. And quantitative analysis of total length and the number of nudes in TPPU + and TPPU- group. *P < 0.05, **P < 0.01, ***P < 0.001

Studies have demonstrated that TPPU can promote the angiogenic effect of HUVECs. While DPSCs are selected as the preferred stem cells for pulp regeneration due to their multidirectional differentiation potential and odontogenic characteristics. Therefore, we first examined the effects of TPPU on proliferation, migration and angiogenesis of HUVECs and DPSCs, respectively. CCK-8 assay showed that TPPU could improve HUVEC proliferation while not affect DPSCs (Fig. 2a, b). Then, the scratch assay and tube formation assay showed that TPPU effectively potentiated wound healing (*P* < 0.001) and tube formation of HUVECs (*P* < 0.05), but had no significant effect on DPSCs (Fig. 2c–f). Several studies have shown that the coculture of endothelial cells (ECs) and DPSCs can promote cell survival, proliferation and migration. We then evaluated the effect of TPPU on co-cultured the HUVECs and DPSCs (1:1, cocultured cells), and found that the proliferation of cocultured cells was enhanced, consistent with the previous study, but TPPU did not further promote this effect (Fig. 2g). Transwell migration assay showed that TPPU significantly promoted transmigration of HUVECs to lower surface of the insert membrane, and chemotaxis-based assay demonstrated that TPPU had an additive chemotactic effect in that enhanced the capacity of DPSCs to recruit HUVECs (Fig. 2h, P < 0.05). Scratch assay and tube formation assay also revealed that TPPU promoted the wound healing and angiogenesis of cocultured cells (Fig. 2i, j, P < 0.05). Taken together, these data suggested that TPPU improved the migration and tube formation potential of cocultured cells, and enhanced the ability of DPSCs to attract ECs, which contribute to angiogenesis.

### TPPU activates the VEGF/VEGFR2 signaling pathway to promote angiogenic-odontogenic coupling mediated by crosstalk between HUVECs and DPSCs

**Fig. 3:**
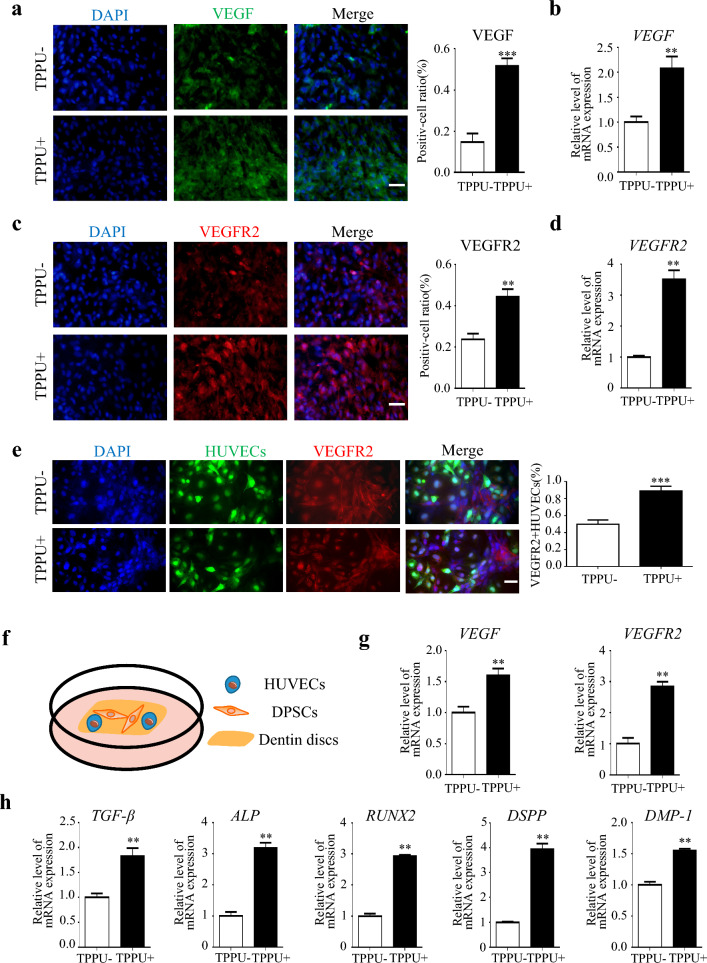
TPPU activates the VEGF/VEGFR2 signaling pathway of cocultured cells to promote angiogenic-odontogenic coupling **a**–**d** IF and RT-qPCR showed the expression of VEGF and VEGFR2. scale bar = 50 µm. **e** Representative IF images of co-cultured HUVECs and DPSCs after 7 days of osteogenic culture for VEGFR2 (red) and HUVECs (GFP-tagged) and quantification of the percentage of VEGFR2 + HUVECs, Scale bar = 100 μm. **f** HUVECs and DPSCs at the ratio of 1:1 were seeded on dentin discs treated with/without TPPU (10 μM) for 7 d. **g**, **h** RT-qPCR showed the gene expression of *VEGF*, *VEGFR2*, *TGF-β, ALP*, *RUNX2*, *DSPP*, and *DMP-1*. **P < 0.01, ***P < 0.001

VEGF/VEGFR2 signaling pathway plays an important role in angiogenesis. A study has shown that VEGFR2 is an important factor for angiogenic-odontogenic coupling around odontoblasts. Therefore, we examined the effect of TPPU on the expression of related factors in coculture cells. In vitro, IF and RT-qPCR showed an increase in VEGF/VEGFR2 expression in TPPU-treated cocultured cells (Fig. 3 a–d, P < 0.01), Western blotting also confirmed that TPPU treatment significantly increased VEGFR2 expression in the cocultured cells (Additional file 2: Fig. S2a). We then further identified that the VEGFR2 was mainly expressed in GFP-tagged HUVECs in cocultured cells (Fig. 3e, P < 0.001), which further suggested that TPPU promoted the crosstalk between cocultured cells by activating the VEGF/VEGFR2 signaling pathway. Next, we established an environment of odontogenic differentiation in the dentin discs and cocultured HUVECs and DPSCs on dentin discs for 7 days (Fig. 3f). RT-qPCR results indicated that TPPU treatment increased the expression of *VEGF* and *VEGFR2* (Fig. 3g, P < 0.01), as well as the expression of osteogenic-odontogenic related gene (*TGF-β, ALP, RUNX2, DSPP, DMP-1*) were upregulated in TPPU-treated cocultured cells compared with the control group (Fig. 3h, P < 0.01), which further suggested that TPPU could enhance angiogenic-odontogenic coupling via VEGF/VEGFR2 pathway.

**Fig. 4:**
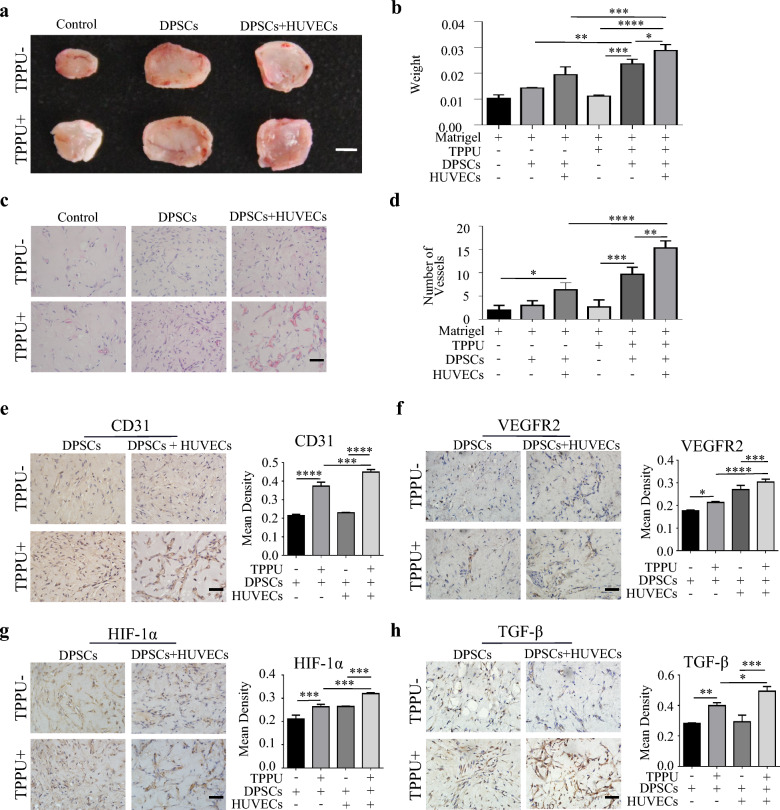
TPPU enhances the formation of VEGFR2 + vessels in grafted Matrigel plug **a** General view of the Matrigel plug-grafts. Scale bar = 250 µm. **b** The weight of grafts in different groups (n = 12/group). **c** Representative histological images of transplanted Matrigel plug. Scale bar = 50 µm. **d** Quantitative analysis of the number of vessels in different groups. **e**, **f** Immunohistochemical staining and quantification showing the expression of CD31 and VEGFR2. Scale bar = 50 µm. **g**, **h** Immunohistochemical staining showing the expression of HIF-1α and TGF-β. Scale bar = 50 µm. **g**, **h** Quantification of HIF-1α and TGF-β expression. *P < 0.05, **P < 0.01, ***P < 0.001, ****P < 0.0001

To further evaluate the effects of TPPU on the crosstalk between HUVECs and DPSCs in vivo, the Matrigel plugs mixed with DPSCs or DPSC + HUVECs were subcutaneously transplanted into BALB/c nude mice, respectively. The isolated grafts at 7 days post-transplantation showed that the TPPU-treated plugs had greater size and weight, and looked more ruddy than those of vehicle treatment (Fig. 4a, b). Consistent with the in vitro results, vascular-like structures were identified in all grafts, especially in TPPU-treated Matrigel plugs (Fig. 4c, d). More CD31, VEGFR2 expressions were detected in TPPU-treated grafts (Fig. 4e, f). There were also more HIF-1α and TGF-β positive cells in TPPU-treated grafts (Fig. 4g, h). Moreover, IHC showed an increased proportion of Ki-67 + cells in TPPU-treated grafts (Additional file 1: Fig. S1a, c), and Tunel staining results showed there were no significant differences between the groups (Additional file 1: Fig. S1b, d). The in vitro and in vivo results further suggested that TPPU enhanced the angiogenic-odontogenic coupling between HUVECs and DPSCs, which was closely related to the activation of HIF-1α, VEGF/VEGFR2 and TGF-β pathway.

### TPPU promotes angiogenic-odontogenic coupling of cocultured cells through HIF-1α

**Fig. 5:**
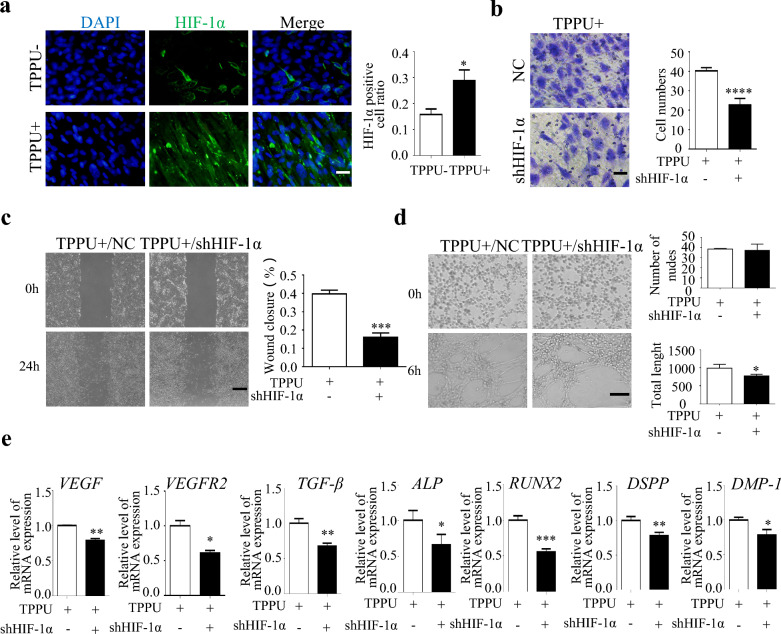
TPPU promotes the POTCs angiogenesis of cocultured cells via HIF-1α **a** IF detection of HIF-1α expression in TPPU- and TPPU + groups. **b** Representative image of Transwell assay and quantitative analysis of the migration cell numbers, scale bar = 50 µm. **c** Representative images of scratch assay and quantitative analysis of the percentage of wound closure, scale bar = 500 µm. **d** Representative images of tube formation assay and quantitative analysis of total length and the number of nudes, scale bar = 100 µm. **e** RT-qPCR showing the expression of *VEGF*, *VEGFR2*, *TGF-β, ALP*, *RUNX2*, *DSPP*, and *DMP-1* in cocultured cells after 7 days of osteogenic induction. *P < 0.05, **P < 0.01, ***P < 0.001, ****P < 0.0001

HIF-1α is an important regulator of the VEGF signaling pathway. IF and Western blotting showed that TPPU treatment significantly increased HIF-1α expression in the cocultured cells (Fig. 5a, Additional file 2: Fig. S2a). Our previous study has shown that TPPU promotes osteogenesis and Type H vessel-related gene expression through increasing HIF-1α levels in HUVECs in the cocultured cells, we further investigated whether HIF-1α influenced the odontogenic fate commitment of coculture cells in this study. When HIF-1α was knockdown (shHIF-1α) in HUVECs in cocultured cells (Additional file 2: Fig. S2b, c)*,* the migration, healing, and tube formation effects enhanced by TPPU were reversed (Fig. 5b–d). The above data suggested that TPPU promoted chemotactic migration and angiogenic capacity of the cocultured cells via HIF-1α. Next, we examined the gene expression changes after 7 d of osteogenic induction by RT-qPCR and Western blotting, and the results showed that shHIF-1α in HUVECs significantly reduced the expression of VEGF, VEGFR2 and osteogenic and odontogenic-related genes compared with the NC group (Fig. 5e, Additional file 2: Fig. S2d). This further suggested that TPPU activated VEGFR2/VEGF signaling pathway and induced osteogenic and odontogenic differentiation through HIF-1α.

### TPPU boosts the formation of VEGFR2 + POTCs and dental pulp regeneration in vivo

**Fig. 6:**
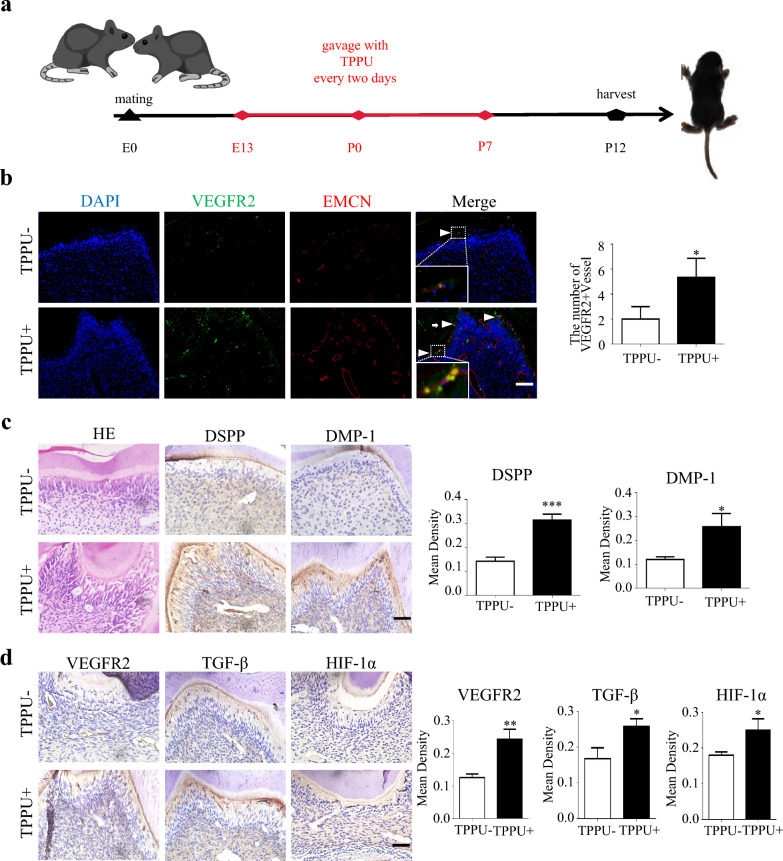
TPPU boosts the formation of VEGFR2 + vessels during the early odontogenesis in molar **a** The flowchart of animal experiment (n = 5/group). **b** IF detection of VEGFR2 + positive vessel (white arrowheads) around odontoblasts in TPPU- and TPPU + groups for VEGFR2 (green) and EMCN (red). Boxed insets showing higher magnification of POTCs with VEGFR2 and EMCN colocalization, scale bar = 100 µm. **c** Representative images of H&E staining and IHC staining for DSPP and DMP-1 in molars, and quantitative analysis. Scale bar = 50 µm. **d** The IHC staining for VEGFR2, TGF-β and HIF-1α in molars and quantitative analysis, scale bar = 50 µm. *P < 0.05, **P < 0.01, ***P < 0.001

VEGFR2 is an important factor in coupling of POTCs and early odontogenesis. We further investigated whether TPPU could promote the formation of POTCs during pulp development by upregulating VEGFR2 in vivo*.* The female mice were gavaged with TPPU from 7 days prepartum through 7 days postpartum, and the lower jaws from pups at postnatal 12 days were isolated for histological staining (Fig. 6a). In the basal layer of odontoblasts, more EMCN + and VEGFR2 + co-localization vessels were seen in the molar of offspring from TPPU-treated female mice (Fig. 6b). H&E staining showed that the TPPU-treated group dentin layer was thicker than the control group (Fig. 6c), and IHC revealed more DSPP, DMP-1, VEGFR2, TGF-β and HIF-1α protein expression in TPPU-treated group (Fig. 6c, d). The same results were obtained in the maxilla, with a higher expression of VEGFR2 + vessels at the odontoblast layer in the TPPU-treated group (Additional file 3: Fig. S3a, b), and higher expression of odontogenesis and angiogenesis-related proteins (Additional file 3: Fig. S3b, c). The above results further confirmed that TPPU promotes angiogenic-odontogenic coupling in the early stage of odontogenesis.

**Fig. 7:**
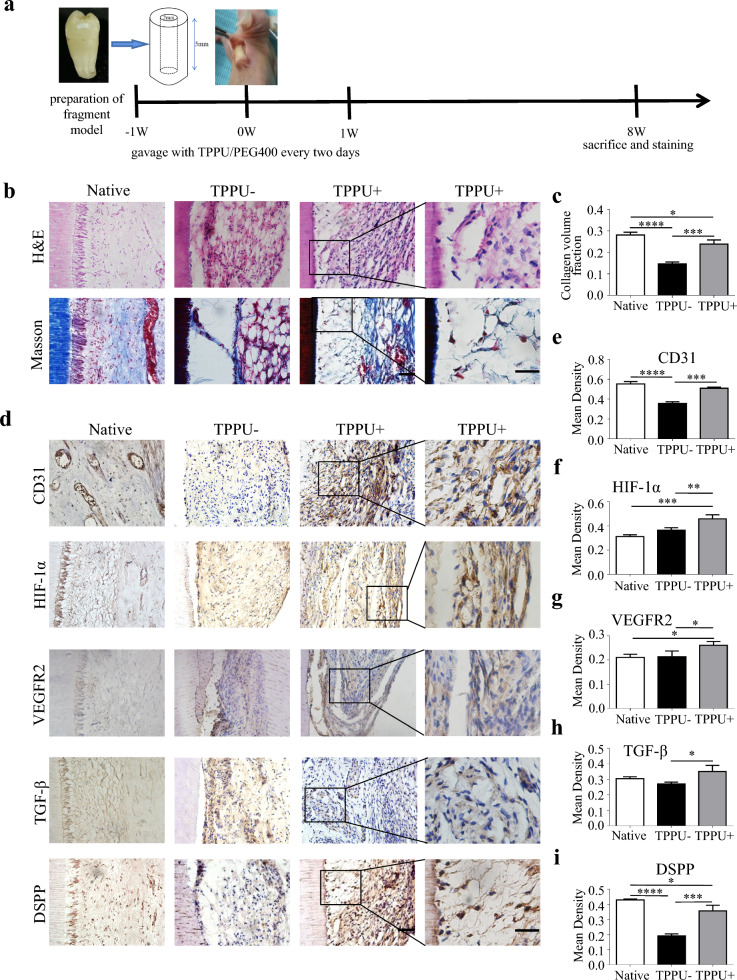
TPPU promotes the formation of pulp-like tissue in root segment transplantation in vivo **a** The flowchart of animal experiment (n = 10/group). **b** Representative H & E and Masson’s trichrome staining images of root canal in Native, TPPU—and TPPU + groups. **c** Quantification of Masson’s trichrome staining in root canal segment. The magnification of boxed areas is shown in right panel. Scale bar = 50 μm (left panel), 25 μm (right panel). **d**–**i** IHC staining and quantification showing the expression of CD31, HIF-1α, VEGFR2, TGF-β, and DSPP. The magnification of boxed areas is shown in right panel. Scale bar = 50 μm (left panel), 25 μm (right panel). *P < 0.05, **P < 0.01, ***P < 0.001, ****P < 0.0001

Based on the above results, we further designed an in vivo root segment transplantation to explore the effect of TPPU on pulp regeneration. After 8 weeks of transplantation of root segments in the mice, pulp tissue, angiogenic and odontogenic differentiation of DPSCs in root canal were measured (Fig. 7a). H & E staining showed that TPPU induced more regenerated pulp-like tissue with higher cell density, blood vessel structure and odontoblast layer near the root canal wall (Fig. 7b). Moreover, Masson's trichrome staining showed that more abundant collagen fibers in regenerated pulp-like tissue in the TPPU treatment group, while no tight junction tissue was found in the root canal wall of the control group (Fig. 7b, c). Furthermore, we evaluated the level of angiogenesis and odontogenesis-related proteins. IHC staining showed that the expression of CD31, HIF-1α, and VEGFR2 were higher in the TPPU + group than in TPPU- group. And TPPU also upregulated the expression of odontogenic markers TGF-β and DSPP in the root canal (Fig. 7d). Quantitative statistics of the above protein expression showed the same trend, while the trend of increase by TPPU in this experiment was not as significant as the above in vivo experiment (Fig. 7e–i). Therefore, these results demonstrated that TPPU triggered the odontogenic capacities of mesenchymal stem cells through revascularization for pulp regeneration, mainly acting on the early stages of odontogenesis via HIF-1α/VEGFR2/ TGF-β pathway.

## Discussion

Dental pulp tissue plays an important role in maintaining tooth health. Due to the peculiarities of immature permanent teeth and deciduous teeth, it is vital to preserve pulp vitality whenever possible [27, 28]. The traditional root canal treatment such as apical induction and promoting the formation of local tooth tissue cannot achieve physiological pulp tissue regeneration. Revascularization as an emerging technique for restoring root development, long-term tooth retention of immature permanent teeth and deciduous teeth has made a lot of progress. In recent years, tissue engineering technology for dental pulp regeneration has offered new options for obtaining pulp vitality to maximize the preservation of tooth function [29]. In our study, we focus on the effects of TPPU on pulp regeneration from two perspectives: early vascularization and odontoblast differentiation. Our results demonstrated that TPPU effectively promotes the coupling of VEGFR2 positive vessel formation and DPSCs odontogenic differentiation, which provides a great potential strategy for tissue-engineered pulp regeneration.

Based on the high-performance properties of TPPU compounds, some studies have also focused on the safe dose and systemic effects of TPPU. Research shows that administration of 3 mg/kg of TPPU to mice can effectively inhibit sEH expression in the hippocampus, striatum, liver, spinal cord, kidneys, and elevate the level of EET [30, 31]. This dose does not produce biotoxicity in the animals, and does not affect diet, water intake, and body weight of the mice, as well as lead to a decrease of systemic inflammatory factors, such as TNF-α and IL-1β. We monitored the body weight of the pregnant mice and neonatal mice treated with TPPU and found no significant differences compared with the control, which suggested that 3 mg/kg dose was safe and effective. Clinical trial of sEH inhibitors, AR9281, GSK2256294 also has provided evidence of safety, no serious adverse events associated with the sEH inhibitors and a potential prospect of clinical application [32, 33].

Based on our experiment, it is difficult to achieve local application of drugs due to the limitations of the pulp-dentin complex, and local stem cell modification is more feasible through systemic delivery. We chose to administer TPPU by gavage to achieve a systemic circulation of TPPU to explore its effects on stem cells implanted within the root canal. We hope to promote regeneration through local stem cell modulation by TPPU, as well as to explore the developmental effects of TPPU by inhibiting sEH protein expression throughout the body. The results of our ex vivo and in vivo studies tentatively support the conjecture that TPPU can promote pulpal regeneration. However, more pharmacological evaluation is necessary to determine the safest and most appropriate dose of TPPU for pulpal regeneration. In addition, we will continue to explore the feasibility of localized application of TPPU for pulp regeneration to further promote the clinical translation of sEH inhibitors.

Dental pulp microvessels play an important role in maintaining cellular self-renewal and tissue viability by transporting nutrients and removing metabolites [34]. Angiogenesis promotes cell survival and tissue regeneration in tissue-engineered dental pulp tissue by providing essential oxygen and nutrients [35, 36]. Studies have shown that EETs play a significant role in promoting microangiogenesis. Increasing EETs levels through genetic modification significantly increased the number of microvessels in liver and lung tissues [37]. EETs effectively ameliorate cerebral ischemia by increasing the microcirculation of brain tissue and improving the nutritional supply of brain nerves [38, 39]. Our in vitro experiments indicated that TPPU enhanced the migration and tubule formation potentials of cocultured DPSCs and ECs.

In odontoblasts, TGF-β plays an important role in the transcriptional regulation of two non-collagenous proteins, DSPP and DMP-1 [40]. Previous studies have shown that liposome-loaded TGF-β1 treatment of hDPSCs significantly increases the expression of "bone dentin" markers of RUNX-2, DMP-1, and DSPP, and the accumulation of mineralized nodules. In our study, TGF-β antibody blockade partially attenuated the promoting effect of TPPU on the expression of DMP-1, and DSPP in vitro, indicating that TGF-β also plays a crucial role in the process of TPPU promoting the angiogenic-odontogenic coupling. However, the relative contributions of TPPU to TGF-β remained unknown.

VEGF secretion and multiple VEGF receptors are essential for the angiogenic process during development. A study has identified a new type of vessel around odontoblasts with strong filamentous pseudopod extension and high expression of VEGFR1 and VEGFR2 (also known as POTCs). The odontoblasts and POTCs are coupled with positive feedback where odontoblasts enhance angiogenic POTCs by secreting VEGF to interact with VEGFR2 of POTCs, and then POTCs, in turn, provide oxygen, phosphate, and odontogenic factors necessary to support maturation and dentinogenesis of odontoblasts [18]. In this study, we confirmed that TPPU significantly increased the expression of VEGFR2 in ECs to promote odontogenic differentiation.

Studies have reported that EETs can enhance angiogenesis and regulate inflammation through a significant elevation of VEGF and TGF-β in wound healing under hypoxic conditions [41]. Our in vitro results suggested that HIF-1α plays a pivotal role in POTCs. Recently, we have demonstrated that TPPU increases HIF-1α levels in cocultured HUVECs and hDPSCs by activating SLIT3 in hDPSCs [13]. HIF-1α, as a transcription factor, could regulate the expression of VEGF and TGF-β [42, 43]. Here, we further showed that TPPU-upregulated HIF-1α is also involved in odontogenesis and angiogenesis with increased expression of VEGFR2 in ECs and VEGF, TGF-β, DSPP, and DMP-1 in DPSCs. Moreover, stabilizing HIF-1α levels in exfoliated dental papilla stem cells enhances cell survival and proliferation and promotes odontoblast-oriented differentiation of stem cells [44, 45]. TPPU up-regulating HIF-1α contributes to improving endothelial cell migration and angiogenesis, implying the potential of TPPU for pulp regeneration, especially in the treatment of immature permanent and deciduous teeth. However, the specific molecular mechanism by which TPPU regulates VEGF/VEGFR2 and TGF-β through HIF-1α in pulp regeneration still needs further exploration.

TGF-β plays diverse roles in angiogenesis and odontogenic differentiation of DPSCs. It has been reported that the secretion of TGF-β1 and BMP is increased after dentin damage which is related to the structural formation of reactive dentin [46]. A study has shown that TGF-β1 treatment induces VEGFR1 expression in endothelial cells but has no significant effect on VEGFR2 expression [47]. However, another study has confirmed that TGF-β1 treatment of endothelial cells increases the release of full-length VEGFR2 protein in the conditioned medium [48]. Although our results suggest that TGF-β is associated with odontoblast differentiation, its relationship with VEGFR2 remains to be further investigated.

## Limitations of the study

While our study demonstrated the roles of a TPPU on POTCs for pulp regeneration in mice, it remains to be seen whether TPPU orchestrating tooth mineralization by coupling angiogenesis and odontogenesis is used for pulp capping and whether topical application of TPPU is also effective. While we showed for the first time the role of TPPU on POTCs, how detailed pathways orchestrate HUVECs and DPSCs remains unclear. Finally, we need to further determine therapeutic dose and side effects of the TPPU.

## Conclusions

Regeneration of dental pulp tissue through tissue engineering has undoubtedly great advantages compared with the current irreversible treatment methods. Our study revealed that TPPU promotes migration and angiogenesis of HUVECs, as well as odonto-osteogenic differentiation of DPSCs. The effects of TPPU enhancing the dentin-pulp complex regeneration mediated by crosstalk between vascular endothelial cells and dental pulp stem cells provide a promising therapeutic application for dental pulp regeneration.

### Supplementary Information

**Additional file 1: Fig. S1.** The effect of TPPU on proliferation and apoptosis in vivo **a**, **c** IHC staining showing the expression of Ki-67, scale bar = 50 µm. And quantification analysis of Ki-67 expression. **b**, **d** Representative images and quantification of TUNEL staining of the transplanted Matrigel plugs, scale bar = 50 µm. *P＜0.05, **P＜0.01, *** *P*＜0.001.

**Additional file 2: Fig. S2.** TPPU regulates the TGF-β, VEGFR2 expression by upregulating HIF-1α in endothelial cells **a** Western blotting showing the protein level of TGF-β, VEGFR2, HIF-1α and quantitative analysis in TPPU- and TPPU+ groups. **b**, **c** RT-qPCR and Western blotting showing the knockdown of HIF-1α in endothelial cells. **d** Knockdown of HIF-1α in endothelial cells, Western blotting showing the protein level of TGF-β, VEGFR2 and quantitative analysis in NC and shHIF-1α group. *P＜0.05, **P＜0.01, *** *P*＜0.001, ****P＜0.0001.

**Additional file 3: Fig. S3.** TPPU boosts the formation of VEGFR2+ vessels in the maxillary molars of C57BL/6 mice **a** IF detection of VEGFR2+ positive vessel (white arrowheads) around odontoblasts in TPPU- and TPPU+ groups for VEGFR2 (green) and EMCN (red). Boxed insets showing higher magnification of POTCs with VEGFR2 and EMCN colocalization, scale bar = 100 µm. **b** Representative images of H and E staining and IHC staining for DSPP and DMP-1 of maxillary molars and quantitative analysis. Scale bar = 50 µm. **c** The IHC staining for VEGFR2, TGF-β and HIF-1α in maxillary molars and quantitative analysis, scale bar = 50 µm. *P＜0.05, **P＜0.01.

**Additional file 4**: **Table S1**. Primers for RT-qPCR. **Table S2.** Target sequence of siRNA.

## Data Availability

All data generated or analyzed during this study are included in this published article [and its Additional files].

## Abbreviations

AA: Arachidonic acid
CCK8: Cell counting Kit 8
CYP: Cytochrome P450
DPSCs: Dental pulp stem cells
DSPP: Dentin sialophosphoprotein
ECs: Endothelial cells
EETs: Epoxyeicosatrienoic acids
H&E: Histological hematoxylin and eosin
HUVECs: Human umbilical vein endothelial cells
IF: Immunofluorescence
IHC: Immunohistochemistry
MSCs: Mesenchymal stem cells
POTCs: Periodontal tip-like endothelial cells subtype
RT-qPCR: Real-time quantitative reverse transcription polymerase chain reaction
TPPU: 1-trifluoromethoxyphenyl-3-(1-propionylpiperidin-4-yl) urea
VEGF: Vascular endothelial growth factor

## References

[1] Sui B, Chen C, Kou X, Li B, Xuan K, Shi S, Jin Y (2019). Pulp stem cell-mediated functional pulp regeneration. J Dent Res.

[2] Yang J, Yuan G, Chen Z (2016). Pulp regeneration: current approaches and future challenges. Front Physiol.

[3] Estrela C, Pécora JD, Estrela CRA, Guedes OA, Silva BSF, Soares CJ, Sousa-Neto MD (2017). Common operative procedural errors and clinical factors associated with root canal treatment. Braz Dent J.

[4] Miran S, Mitsiadis TA, Pagella P (2016). Innovative dental stem cell-based research approaches: the future of dentistry. Stem Cells Int.

[5] Lin J, Zeng Q, Wei X, Zhao W, Cui M, Gu J, Lu J, Yang M, Ling J (2017). Regenerative endodontics versus apexification in immature permanent teeth with apical periodontitis: a prospective randomized controlled study. J Endod.

[6] Spector AA, Fang X, Snyder GD, Weintraub NL (2004). Epoxyeicosatrienoic acids (EETs): metabolism and biochemical function. Prog Lipid Res.

[7] Sacerdoti D, Pesce P, Di Pascoli M, Bolognesi M (2016). EETs and HO-1 cross-talk. Prostaglandins Other Lipid Mediat.

[8] Pozzi A, Macias-Perez I, Abair T, Wei S, Su Y, Zent R, Falck JR, Capdevila JH (2005). Characterization of 5,6- and 8,9-epoxyeicosatrienoic acids (5,6- and 8,9-EET) as potent in vivo angiogenic lipids. J Biol Chem.

[9] Alkayed NJ, Cao Z, Qian ZY, Nagarajan S, Liu X, Nelson JW, Xie F, Li B, Fan W, Liu L (2022). Control of coronary vascular resistance by eicosanoids via a novel GPCR. Am J Physiol Cell Physiol.

[10] Wang L, Luo G, Zhang L, Geng H (2018). Neuroprotective effects of epoxyeicosatrienoic acids. Prostaglandins Other Lipid Mediat.

[11] Liu L, Puri N, Raffaele M, Schragenheim J, Singh SP, Bradbury JA, Bellner L, Vanella L, Zeldin DC, Cao J (2018). Ablation of soluble epoxide hydrolase reprogram white fat to beige-like fat through an increase in mitochondrial integrity, HO-1-adiponectin in vitro and in vivo. Prostaglandins Other Lipid Mediat.

[12] Panigrahy D, Kalish BT, Huang S, Bielenberg DR, Le HD, Yang J, Edin ML, Lee CR, Benny O, Mudge DK (2013). Epoxyeicosanoids promote organ and tissue regeneration. Proc Natl Acad Sci USA.

[13] Gao L, Chen W, Li L, Li J, Kongling W, Zhang Y, Yang X, Zhao Y, Bai J, Wang F (2023). Targeting soluble epoxide hydrolase promotes osteogenic-angiogenic coupling via activating SLIT3/HIF-1α signalling pathway. Cell Prolif.

[14] Murray PE, Garcia-Godoy F, Hargreaves KM (2007). Regenerative endodontics: a review of current status and a call for action. J Endod.

[15] Hicklin DJ, Ellis LM (2005). Role of the vascular endothelial growth factor pathway in tumor growth and angiogenesis. J Clin Oncol.

[16] Salati M, Caputo F, Bocconi A, Cerri S, Baldessari C, Piacentini F, Dominici M, Gelsomino F (2022). Successes and failures of angiogenesis blockade in gastric and gastro-esophageal junction adenocarcinoma. Front Oncol.

[17] Dvorak HF (2002). Vascular permeability factor/vascular endothelial growth factor: a critical cytokine in tumor angiogenesis and a potential target for diagnosis and therapy. J Clin Oncol.

[18] Matsubara T, Iga T, Sugiura Y, Kusumoto D, Sanosaka T, Tai-Nagara I, Takeda N, Fong GH, Ito K, Ema M (2022). Coupling of angiogenesis and odontogenesis orchestrates tooth mineralization in mice. J Exp Med.

[19] Dong L, Zhou Y, Zhu ZQ, Liu T, Duan JX, Zhang J, Li P, Hammcok BD, Guan CX (2017). soluble epoxide hydrolase inhibitor suppresses the expression of triggering receptor expressed on myeloid cells-1 by inhibiting NF-kB activation in murine macrophage. Inflammation.

[20] Trindade-da-Silva CA, Clemente-Napimoga JT, Abdalla HB, Rosa SM, Ueira-Vieira C, Morisseau C, Verri WA, Montalli VAM, Hammock BD, Napimoga MH (2020). Soluble epoxide hydrolase inhibitor, TPPU, increases regulatory T cells pathway in an arthritis model. Faseb j.

[21] Zhou Y, Yang J, Sun GY, Liu T, Duan JX, Zhou HF, Lee KS, Hammock BD, Fang X, Jiang JX (2016). Soluble epoxide hydrolase inhibitor 1-trifluoromethoxyphenyl-3- (1-propionylpiperidin-4-yl) urea attenuates bleomycin-induced pulmonary fibrosis in mice. Cell Tissue Res.

[22] Ulu A, Appt S, Morisseau C, Hwang SH, Jones PD, Rose TE, Dong H, Lango J, Yang J, Tsai HJ (2012). Pharmacokinetics and in vivo potency of soluble epoxide hydrolase inhibitors in cynomolgus monkeys. Br J Pharmacol.

[23] Hu S, Chen B, Zhou J, Liu F, Mao T, Pathak JL, Watanabe N, Li J (2023). Dental pulp stem cell-derived exosomes revitalize salivary gland epithelial cell function in NOD mice via the GPER-mediated cAMP/PKA/CREB signaling pathway. J Transl Med.

[24] Widbiller M, Driesen RB, Eidt A, Lambrichts I, Hiller KA, Buchalla W, Schmalz G, Galler KM (2018). Cell homing for pulp tissue engineering with endogenous dentin matrix proteins. J Endod.

[25] Park YH, Lee YS, Seo YM, Seo H, Park JS, Bae HS, Park JC (2020). Midkine promotes odontoblast-like differentiation and tertiary dentin formation. J Dent Res.

[26] Bai J, Li L, Kou N, Bai Y, Zhang Y, Lu Y, Gao L, Wang F (2021). Low level laser therapy promotes bone regeneration by coupling angiogenesis and osteogenesis. Stem Cell Res Ther.

[27] Swaikat M, Faus-Matoses I, Zubizarreta-Macho Á, Ashkar I, Faus-Matoses V, Bellot-Arcís C, Iranzo-Cortés JE, Montiel-Company JM (2023). Is revascularization the treatment of choice for traumatized necrotic immature teeth? A systematic review and meta-analysis. J Clin Med.

[28] Xuan K, Li B, Guo H, Sun W, Kou X, He X, Zhang Y, Sun J, Liu A, Liao L (2018). Deciduous autologous tooth stem cells regenerate dental pulp after implantation into injured teeth. Sci Transl Med.

[29] Zhai Q, Dong Z, Wang W, Li B, Jin Y (2019). Dental stem cell and dental tissue regeneration. Front Med.

[30] Luo A, Wu Z, Li S, McReynolds CB, Wang D, Liu H, Huang C, He T, Zhang X, Wang Y (2023). The soluble epoxide hydrolase inhibitor TPPU improves comorbidity of chronic pain and depression via the AHR and TSPO signaling. J Transl Med.

[31] Ren Q, Ma M, Ishima T, Morisseau C, Yang J, Wagner KM, Zhang JC, Yang C, Yao W, Dong C (2016). Gene deficiency and pharmacological inhibition of soluble epoxide hydrolase confers resilience to repeated social defeat stress. Proc Natl Acad Sci U S A.

[32] Chen D, Whitcomb R, MacIntyre E, Tran V, Do ZN, Sabry J, Patel DV, Anandan SK, Gless R, Webb HK (2012). Pharmacokinetics and pharmacodynamics of AR9281, an inhibitor of soluble epoxide hydrolase, in single- and multiple-dose studies in healthy human subjects. J Clin Pharmacol.

[33] Lazaar AL, Yang L, Boardley RL, Goyal NS, Robertson J, Baldwin SJ, Newby DE, Wilkinson IB, Tal-Singer R, Mayer RJ (2016). Pharmacokinetics, pharmacodynamics and adverse event profile of GSK2256294, a novel soluble epoxide hydrolase inhibitor. Br J Clin Pharmacol.

[34] Dissanayaka WL, Zhang C (2017). The role of vasculature engineering in dental pulp regeneration. J Endod.

[35] Xu X, Liang C, Gao X, Huang H, Xing X, Tang Q, Yang J, Wu Y, Li M, Li H (2021). Adipose Tissue-derived microvascular fragments as vascularization units for dental pulp regeneration. J Endod.

[36] Saghiri MA, Asatourian A, Sorenson CM, Sheibani N (2015). Role of angiogenesis in endodontics: contributions of stem cells and proangiogenic and antiangiogenic factors to dental pulp regeneration. J Endod.

[37] Webler AC, Michaelis UR, Popp R, Barbosa-Sicard E, Murugan A, Falck JR, Fisslthaler B, Fleming I (2008). Epoxyeicosatrienoic acids are part of the VEGF-activated signaling cascade leading to angiogenesis. Am J Physiol Cell Physiol.

[38] Liu Z, Liu Y, Zhou H, Fu X, Hu G (2017). Epoxyeicosatrienoic acid ameliorates cerebral ischemia-reperfusion injury by inhibiting inflammatory factors and pannexin-1. Mol Med Rep.

[39] Qu YY, Yuan MY, Liu Y, Xiao XJ, Zhu YL (2015). The protective effect of epoxyeicosatrienoic acids on cerebral ischemia/reperfusion injury is associated with PI3K/Akt pathway and ATP-sensitive potassium channels. Neurochem Res.

[40] Niwa T, Yamakoshi Y, Yamazaki H, Karakida T, Chiba R, Hu JC, Nagano T, Yamamoto R, Simmer JP, Margolis HC (2018). The dynamics of TGF-β in dental pulp, odontoblasts and dentin. Sci Rep.

[41] Sommer K, Jakob H, Badjlan F, Henrich D, Frank J, Marzi I, Sander AL (2019). 11,12 and 14,15 epoxyeicosatrienoic acid rescue deteriorated wound healing in ischemia. PLoS ONE.

[42] Shi Y, Wang S, Zhang W, Zhu Y, Fan Z, Huang Y, Li F, Yang R (2022). Bone marrow mesenchymal stem cells facilitate diabetic wound healing through the restoration of epidermal cell autophagy via the HIF-1α/TGF-β1/SMAD pathway. Stem Cell Res Ther.

[43] Wan C, Gilbert SR, Wang Y, Cao X, Shen X, Ramaswamy G, Jacobsen KA, Alaql ZS, Eberhardt AW, Gerstenfeld LC (2008). Activation of the hypoxia-inducible factor-1alpha pathway accelerates bone regeneration. Proc Natl Acad Sci USA.

[44] Han Y, Koohi-Moghadam M, Chen Q, Zhang L, Chopra H, Zhang J, Dissanayaka WL (2022). HIF-1α stabilization boosts pulp regeneration by modulating cell metabolism. J Dent Res.

[45] Efimenko A, Starostina E, Kalinina N, Stolzing A (2011). Angiogenic properties of aged adipose derived mesenchymal stem cells after hypoxic conditioning. J Transl Med.

[46] de Oliveira Luiz, da Rosa W, Machado da Silva T, Fernando Demarco F, Piva E, Fernandes da Silva A (2017). Could the application of bioactive molecules improve vital pulp therapy success? A systematic review. J Biomed Mater Res A.

[47] Shih SC, Ju M, Liu N, Mo JR, Ney JJ, Smith LE (2003). Transforming growth factor beta1 induction of vascular endothelial growth factor receptor 1: mechanism of pericyte-induced vascular survival in vivo. Proc Natl Acad Sci U S A.

[48] Jarad M, Kuczynski EA, Morrison J, Viloria-Petit AM, Coomber BL (2017). Release of endothelial cell associated VEGFR2 during TGF-β modulated angiogenesis in vitro. BMC Cell Biol.

