# Supplementary figures and images for: Inhibition of soluble epoxide hydrolase enhances the dentin-pulp complex regeneration mediated by crosstalk between vascular endothelial cells and dental pulp stem cells
Source: J Transl Med. 2024 Jan 16;22:61. doi: 10.1186/s12967-024-04863-y (PMC10790489; doi:10.1186/s12967-024-04863-y)

**a**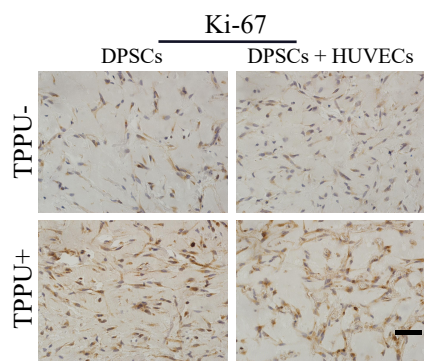**c**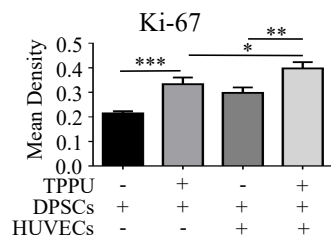**b**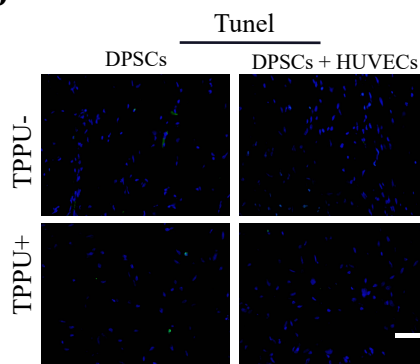**d**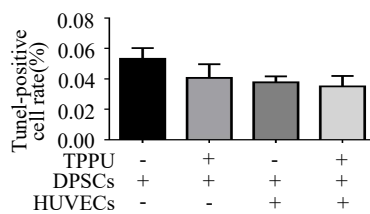

Supplement: Supplementary file 1 — Additional file 1: Fig. S1. The effect of TPPU on proliferation and apoptosis in vivo a, c IHC staining showing the expression of Ki-67, scale bar = 50 µm. And quantification analysis of Ki-67 expression. b, d Representative images and quantification of TUNEL staining of the transplanted Matrigel plugs, scale bar = 50 µm. *P＜0.05, **P＜0.01, *** P＜0.001. [file 12967_2024_4863_MOESM1_ESM.pdf]

**a**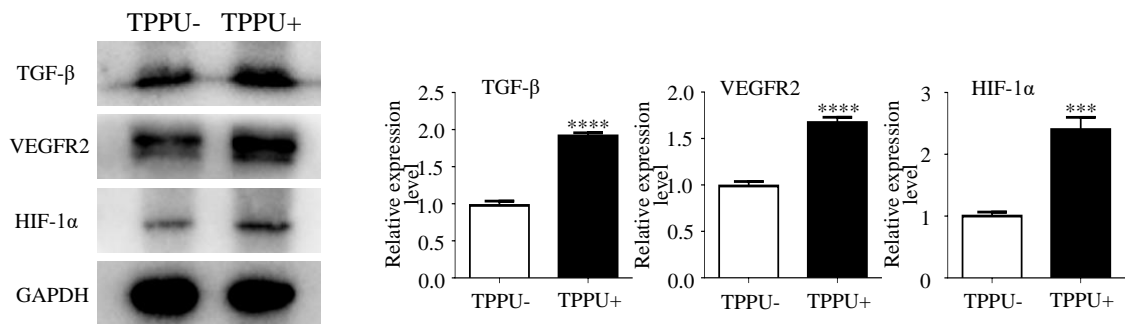**b**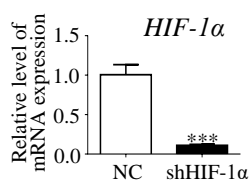**c**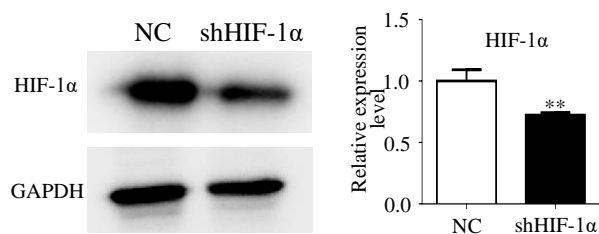**d**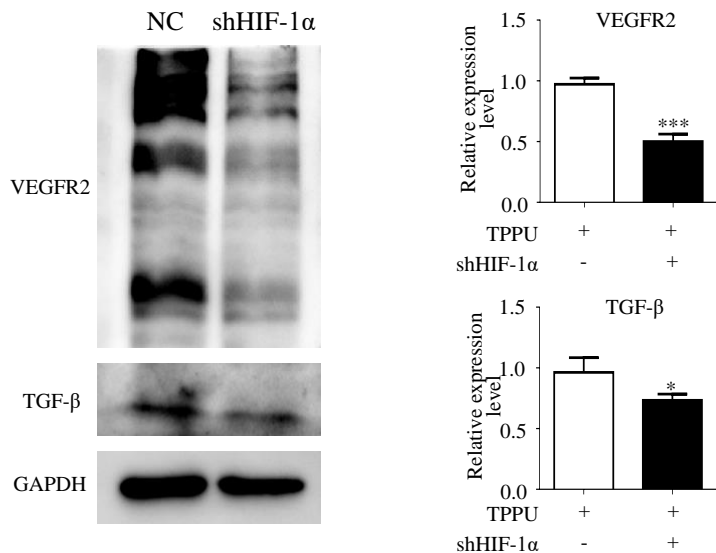

Supplement: Supplementary file 2 — Additional file 2: Fig. S2. TPPU regulates the TGF-β, VEGFR2 expression by upregulating HIF-1α in endothelial cells a Western blotting showing the protein level of TGF-β, VEGFR2, HIF-1α and quantitative analysis in TPPU- and TPPU+ groups. b, c RT-qPCR and Western blotting showing the knockdown of HIF-1α in endothelial cells. d Knockdown of HIF-1α in endothelial cells, Western blotting showing the protein level of TGF-β, VEGFR2 and quantitative analysis in NC and shHIF-1α group. *P＜0.05, **P＜0.01, *** P＜0.001, ****P＜0.0001. [file 12967_2024_4863_MOESM2_ESM.pdf]

**a**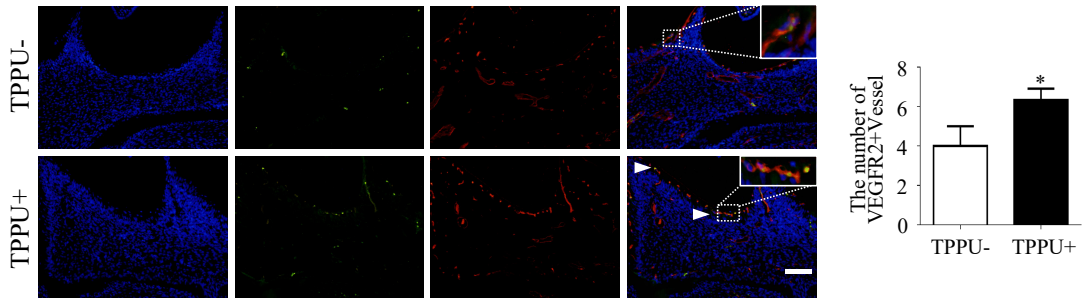**b**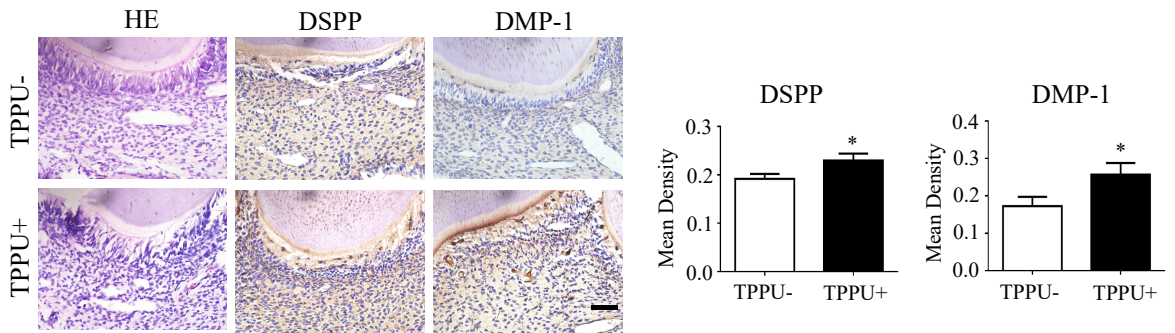**c**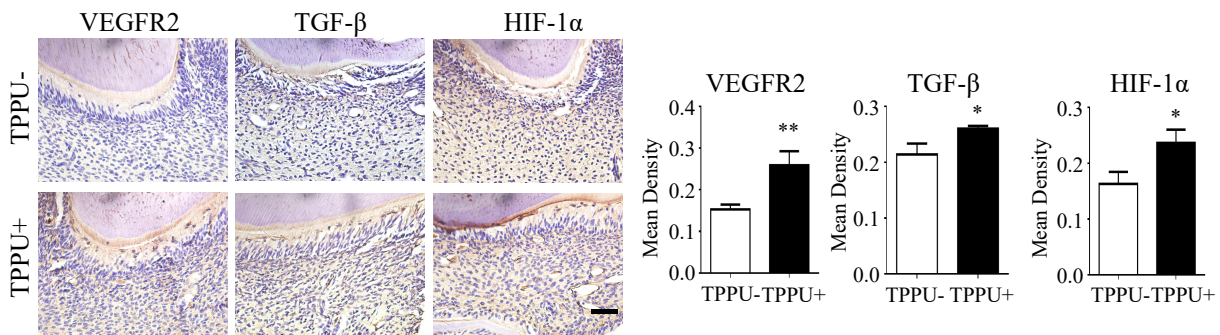

Supplement: Supplementary file 3 — Additional file 3: Fig. S3. TPPU boosts the formation of VEGFR2+ vessels in the maxillary molars of C57BL/6 mice a IF detection of VEGFR2+ positive vessel (white arrowheads) around odontoblasts in TPPU- and TPPU+ groups for VEGFR2 (green) and EMCN (red). Boxed insets showing higher magnification of POTCs with VEGFR2 and EMCN colocalization, scale bar = 100 µm. b Representative images of H and E staining and IHC staining for DSPP and DMP-1 of maxillary molars and quantitative analysis. Scale bar = 50 µm. c The IHC staining for VEGFR2, TGF-β and HIF-1α in maxillary molars and quantitative analysis, scale bar = 50 µm. *P＜0.05, **P＜0.01. [file 12967_2024_4863_MOESM3_ESM.pdf]
