# Supplementary material for: Inhibition of soluble epoxide hydrolase enhances the dentin-pulp complex regeneration mediated by crosstalk between vascular endothelial cells and dental pulp stem cells
Source: J Transl Med. 2024 Jan 16;22:61. doi: 10.1186/s12967-024-04863-y (PMC10790489; doi:10.1186/s12967-024-04863-y)
Supplement: Supplementary file 4 — Additional file 4: Table S1. Primers for RT-qPCR. Table S2. Target sequence of siRNA. [file 12967_2024_4863_MOESM4_ESM.docx]

**Additional file Tables**

**Table S1. Primers for RT-qPCR**

| Primers | Forward primer (5’-3’) | Reverse primer(5’-3’) |
| --- | --- | --- |
| *ALP* | AGGAACGGATCTCGGGGTA | TGGGGTCTTTCTCTTTCTCTGG |
| *DMP-1* | CCTGAGGATGAGAACAGCTCCA | GATCTGCTGCTGTCTTGAGAGTCAC |
| *DSPP* | CCAGAGCAAGTCTGGTAACGGTAA | GTCACTGCCTTCACTGTCACTGTC |
| *GAPDH* | AGCACCGTCAAGGCTGAGAAC | TGGTGAAGACGCCAGTGGA |
| *HIF-1α* | ACCGCTGAAACGCCAAAG | TCCATCGGAAGGACTAGGTGTCT |
| *RUNX2* | CATGGCCGGGAATGATGAG | TGTGAAGACCGTTATGGTCAAAGTG |
| *TGF-β* | CGCCAGAGTGGTTATCTTTTG | CGGTAGTGAACCCGTTGATGT |
| *VEGF* | AGGAGTACCCTGATGAGATCGAGT | TGGTGAGGTTTGATCCGCATA |
| *VEGFR2* | CTCTTGGCCGTGCCTTTG | GTGTGTTGCTCCTTCTTTCAAC |

**Table S2. Target sequence of siRNA**

| siRNA target genes | Target sequence |
| --- | --- |
| shHIF-1α | 5’- CCAGCAGACUCAAAUACAATT -3’ |
| NC | 5’- UUCUCCGAACGUGUCACGUTT -3’ |
